# Increasing incidence of invasive nontyphoidal *Salmonella* infections in Queensland, Australia, 2007-2016

**DOI:** 10.1371/journal.pntd.0007187

**Published:** 2019-03-18

**Authors:** Andrea Parisi, John A. Crump, Russell Stafford, Kathryn Glass, Benjamin P. Howden, Martyn D. Kirk

**Affiliations:** 1 National Centre for Epidemiology and Population Health, Australian National University, Canberra, Australia; 2 Centre for International Health, University of Otago, Dunedin, New Zealand; 3 Communicable Diseases Unit, Queensland Health, Brisbane, Australia; 4 Microbiological Diagnostic Unit Public Health Laboratory, University of Melbourne, Melbourne, Australia; The University of Sheffield, UNITED KINGDOM

## Abstract

Nontyphoidal *Salmonella* is a major contributor to the global burden of foodborne disease, with invasive infections contributing substantially to illnesses and deaths. We analyzed notifiable disease surveillance data for invasive nontyphoidal *Salmonella* disease (iNTS) in Queensland, Australia. We used Poisson regression to estimate incidence rate ratios by gender, age group, and geographical area over 2007–2016. There were 995 iNTS cases, with 945 (92%) confirmed by blood culture. *Salmonella* Virchow accounted for 254 (25%) of 1,001 unique iNTS isolates. Invasive NTS disease notification rates peaked among infants, during the summer months, and in outback Queensland where the notification rate (95% CI) was 17.3 (14.5–20.1) cases per 100,000 population. Overall, there was a 6,5% annual increase (p<0.001) in iNTS disease incidence. In conclusion, high iNTS rates among males, infants, and the elderly require investigation of household level risk factors for NTS infection. Controlling *Salmonella* Virchow infections is a public health priority.

## Author summary

We identified increasing incidence of invasive infections due to nontyphoidal *Salmonella* in Queensland with particularly high rates of disease among males, infants, elderly people, and cases infected with *Salmonella* serotype Virchow. *Salmonella* serotypes Choleraesuis, Dublin, and Panama had the highest proportion of invasive isolates.

## Introduction

Nontyphoidal *Salmonella* (NTS) infections are a serious public health concern globally. In high-income settings, NTS predominantly causes a self-limiting diarrhoeal illness with low case fatality risk [1]. NTS may also invade beyond the gastrointestinal tract and cause severe disease, including bacteremia and infection of other normally sterile sites referred to as invasive nontyphoidal *Salmonella* (iNTS) disease. Children under the age of five years, the elderly, those with comorbid illnesses, and the immunocompromised are at particular risk of developing iNTS disease [2,3]. Invasive disease caused by NTS is particularly common in low-resource settings with high prevalence of HIV, malaria, anemia, and malnutrition [4].

The case fatality risk of NTS bacteremia is as high as 19% in sub-Saharan Africa and 26% in Vietnam [2,5]. Due to its severity and health impact, there have been several attempts to estimate the global burden of iNTS disease since 2010. The study of Ao et al. estimated 3.4 million cases of iNTS disease resulting in 681,316 deaths annually [6]. Another study, which unlike Ao et al. excluded HIV-associated iNTS disease, estimated 596,824 cases of iNTS accounting for 63,312 deaths in 2010 [7]. However, both estimates are based on very few studies, therefore, additional data are needed to improve current estimates and obtain a more complete picture of the burden of disease.

In Australia, salmonellosis is one of the most important causes of foodborne diarrheal disease due to its increasing incidence and propensity to cause large outbreaks. Almost all states and territories have experienced increasing rates of NTS infection since 2000 [8]. Tropical areas of Australia experience rates amongst the highest in the industrialised world [9]. In 2016, 18,059 notifications of *Salmonella* infection were reported across Australia at a rate of 74.6 cases per 100,000 population, a 25% increase compared with the mean rate for the previous 5 years. In Queensland, the NTS notification rate was 99.2 cases per 100,000 population in 2016—one of the highest rates in Australia [10]. Recent studies of NTS in the Australian Capital Territory and Victoria found that iNTS comprised 1.6% [11] and 2.5% [12] of all NTS notifications with *Salmonella* Typhimurium being the most commonly reported serotype.

The epidemiology of iNTS disease in Queensland is poorly understood in terms of severity, risk factors, and the distribution of infecting serotypes. Understanding disease incidence and the geographical distribution of iNTS disease is important for effective control of foodborne diseases. In this study, we investigated the descriptive epidemiology of iNTS in Queensland to identify serotypes and high-risk populations to prioritise control measures.

## Materials and methods

We used non-identifiable human illness data on *Salmonella enterica* notifications in Queensland from 2007 through 2016 to analyze trends in disease incidence. Queensland is one of eight Australian states and territories. We chose to analyze iNTS in Queensland due to high rates of salmonellosis overall, varied climate encompassing tropical, subtropical, hot arid, and warm temperate zones, and to include populations living in lower socio-economic areas who might be at greater risk for invasive bacterial infections. Area-level socio-economic indices identify remote parts of Queensland, Townsville, and Cairns as among the most disadvantaged in Australia [13].

### Surveillance system

All Australian states and territories have public health legislation requiring doctors and pathology laboratories to notify any laboratory-confirmed case of salmonellosis [14]. State and territory health departments record details of notified cases on surveillance databases. It is estimated that that for each nontyphoidal *Salmonella* infection, there are 7 undiagnosed cases in the community [15] confirming that notification rates to the surveillance system underestimate disease incidence.

In Queensland, notifiable conditions are reported to Queensland Health and are held in the Notifiable Conditions System (NOCS). Data from NOCS and other databases are aggregated into a national database, the National Notifiable Diseases Surveillance System, operating since 1991 [10].

### Data sources

We obtained non-identified *Salmonella enterica* notifications from NOCS including: ‘notification id,’ ‘person id,’ ‘age at onset,’ ‘onset year,’ ‘onset month,’ ‘diagnosis date,’ ‘collection date,’ ‘hospital and health service,’ ‘postcode,’ ‘locality,’ ‘Statistical Local Area (SLA),’ ‘sex,’ ‘test id,’ ‘specimen,’ ‘serotype,’ ‘subtype,’ and ‘site of infection.’

SLAs have been in use since 1984 as general purpose spatial units covering Australia without gaps or overlaps. There were 475 SLAs in Queensland, defined under The Australian Standard Geographical Classification [16]. In 2011, this system was replaced by The Australian Statistical Geography Standard (ASGS), which has seven hierarchical levels comprising in ascending order: Mesh Block, Statistical Areas Levels 1–4 (SA1-4), State and Territory, and Australia. Each level directly aggregates to the level above [17].

**Fig 1:**
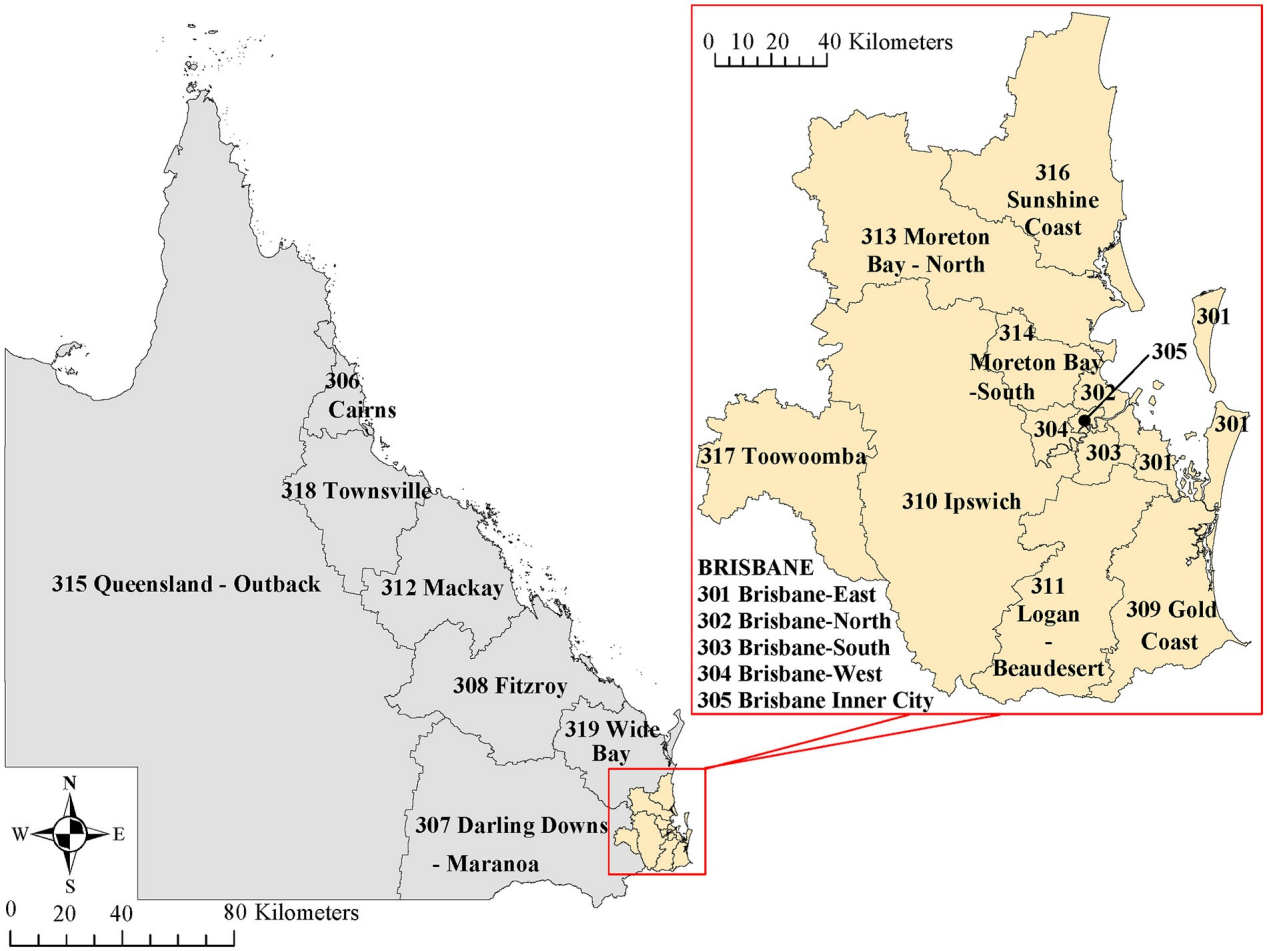
Map of Queensland divided into statistical areas level 4.

For the purposes of our analyses, SLAs were converted into 19 SA4s (Fig 1), the largest sub-State spatial units in the main structure of the ASGS. The conversion was done in several steps. Firstly, SLA was converted into SA2, a medium-sized area built up from whole SA1, and consequently into SA4 using the conversion file from the Australian Bureau of Statistics (ABS) which develops standard statistical geographies and frameworks [18]. Secondly, SLA codes, postcodes, and locations from NOCS were compared and basic checks were done. Lastly, for selected non-matching locations, manual mapping of SLAs and SA4s was performed using the ABS map [19].

We defined an NTS case as a notified infection in a resident of Queensland with a culture-confirmed NTS isolated from any source. If an isolate came from blood, cerebrospinal fluid, peritoneal fluid, pleural fluid, synovial fluid, bone, or other normally sterile site, it was considered as an iNTS case. An individual could meet the case definition more than once if a subsequent infection occurred >30 days after an episode of culture-confirmed NTS infection of a normally sterile site, hereafter referred to as a recurrent iNTS case. If an isolate came from stool, urine, vomitus, sputum, skin, soft tissue abscesses, and wounds, it was defined as a non-invasive NTS case. If an individual had *Salmonella* isolated from the gallbladder only, it was also considered as non-invasive infection due to possible persistence of carriage at that site [20]. Due to possible long-term *Salmonella* shedding in stool [21], we considered recurrent non-invasive NTS cases as those where the period between culture-confirmed episodes of salmonellosis was more than 6 months if caused by the same serotype, and 3 months if serotypes differed.

Cases of *Salmonella enterica* serotype Typhi and serotypes Paratyphi A, B, and C were excluded except for *Salmonella* Paratyphi B biovar Java which predominantly causes enterocolitis rather than paratyphoid fever. *Salmonella* Paratyphi B biovar Java infections were grouped with other NTS serotypes [22,23]. NTS cases residing or having been diagnosed in other states or territories of Australia or abroad and cases with missing information on age, gender, or collection site were excluded from the analysis. Specimens collected post-mortem were excluded from the analysis due to the possibility of bacterial translocation into normally sterile sites after death potentially resulting in misclassification of invasive disease.

We used the date a specimen was collected for all analyses as it was the closest available date to a person’s onset of illness. Phage type data were analyzed only for the two most common iNTS serotypes. In specimen analysis, all normally sterile sites from which iNTS specimens were collected from the same case were analyzed to demonstrate the most common infection site. Otherwise, for the purposes of all analyses, each case was registered once per episode of iNTS infection, regardless of the number of specimens or serotypes per iNTS case.

### Data analyses

We calculated an invasiveness index as the proportion of invasive isolates to the total number of isolates recovered for each serotype. For serotype analysis and invasiveness index calculations, we used the number of unique iNTS isolates as some cases were infected by multiple different serotypes. To determine the effect of age group, gender, geographical area, time, and serotype on invasiveness, we calculated crude odds ratios (OR) and adjusted odds ratios (aOR) comparing invasive and non-invasive NTS infections. The final model was constructed using variables significantly associated with the outcome in the univariable analysis.

In addition, we calculated crude and adjusted incidence rate ratios (IRR) with associated 95% confidence intervals (CI) and p-values by age group, gender, geographical area, and time. Results with p ≤ 0.05 were considered significant. Chi squared tests were used to test for heterogeneity and trends in proportions, two sample t-tests to compare means, and Pearson rank correlations to determine the correlation between the variables and crude incidence rate. For the multivariable analysis of incidence rates, we used a Poisson model, confirming that there was very little overdispersion in the data. Rates of iNTS disease per 100,000 population were estimated using the data on the population residing in Queensland as of the June quarter for each year between 2007 and 2016, obtained from the ABS [24] as the offset in the model.

In the descriptive analysis, invasiveness analysis, and for IRR calculations, age was categorized into 11 age groups: 3 age groups (<1, 1–4, 5–9) until the age of 10 years and then into 10-year age groups until 80 years and over. In the multivariable analysis of incidence rates, ten age groups were included as the first two age groups were combined into one age group (0–4). This combination was necessary due to the unavailability of population data by age, gender, and location from 2007–2010. Age group 30 to 39 years of age was used as a reference category to highlight high-risk age categories. All analyses were performed using Stata version 14 (StataCorp, USA). ArcGIS v10.5 (ESRI, USA) was used to create a map of Australia and Queensland statistical divisions.

### Research ethics

Ethics approval was obtained from the Australian National University Human Research Ethics Committee prior to the conduct of the study [protocol 2017/545]. The Queensland Health data custodian provided approval to access the notifiable disease data.

## Results

There were 32,117 NTS cases reported over the 10-year period in Queensland. Of these, 153 (0.5%) cases were excluded due to following reasons: 136 (88.9%) cases resided or were diagnosed outside Queensland, seven (4.6%) had incomplete information on age and gender, five (3.3%) had isolates collected post-mortem, four (2.6%) had a non-specified specimen source, and one (0.7%) was incorrectly categorized as NTS. Of 31,964 NTS cases, 995 (3.1%) were identified as iNTS and further analyzed.

Among 995 iNTS cases, 13 were infected with two different serotypes per episode of salmonellosis, giving a total of 1008 unique iNTS isolates. Among 1,001 (99.3%) iNTS isolates serotyped, 254 (25.4%) were serotype Virchow, 203 (20.3%) serotype Typhimurium, and 74 (7.4%) serotype Aberdeen. The invasiveness index was 9.06% (254/2804) for serotype Virchow, 2.04% (203/9942) for serotype Typhimurium, and 6.11% (74/1212) for serotype Aberdeen (Table 1). The proportion of iNTS serotypes differed among statistical areas (S1 Table). The most commonly isolated invasive strain of *Salmonella* serotypes Virchow and Typhimurium were phage types 8 and 135A, respectively (S2 and S3 Tables). When compared to serotype Typhimurium, *Salmonella* serotypes Virchow and Aberdeen were associated with invasive disease (aOR, 4.7; 95% CI, 3.9–5.8; and aOR, 2.2; 95% CI, 2.2–3.9, respectively) when adjusted for age group and gender (Table 1). Time, geographical location, and season were not significant predictors for invasive disease. Overall, the odds ratios for invasive disease were highest among males, infants, the elderly (S4 Table), and those infected with *Salmonella* serotypes Choleraesuis, Dublin, and Panama. However, these serotypes represented a small proportion of all iNTS cases (Table 1).

**Table 1:** Distribution and invasiveness of most common iNTS serotypes in Queensland, 2007–2016.

Serotype	No. (%) of iNTS isolates	No. (%) of non-iNTS isolates	Invasive-ness index*	aOR^†^	95% CI		p-value
VIRCHOW	254 (25.37)	2550 (8.76)	9.06	4.74	3.90	5.76	<0.001
TYPHIMURIUM	203 (20.28)	9739 (33.44)	2.04	reference			
ABERDEEN	74 (7.39)	1138 (3.91)	6.11	2.90	2.19	3.85	<0.001
ENTERITIDIS	45 (4.5)	1279 (4.39)	3.40	1.63	1.17	2.27	<0.001
SAINTPAUL	44 (4.4)	2183 (7.50)	1.98	0.97	0.69	1.34	0.84
CHESTER	36 (3.6)	569 (1.95)	5.95	3.08	2.13	4.45	<0.001
BIRKENHEAD	33 (3.3)	1054 (3.62)	3.04	1.34	0.92	1.96	0.13
WAYCROSS	31 (3.1)	677 (2.32)	4.38	2.11	1.42	3.13	<0.001
MGULANI	19 (1.9)	221 (0.76)	7.92	4.25	2.58	7.00	<0.001
JAVIANA	18 (1.8)	236 (0.81)	7.09	3.08	1.86	5.11	<0.001
CORVALLIS	16 (1.6)	266 (0.91)	5.67	2.50	1.47	4.25	0.001
HEIDELBERG	15 (1.5)	143 (0.49)	9.49	5.24	3.00	9.16	<0.001
SUBSPECIES 1	13 (1.3)	431 (1.48)	2.93	1.32	0.74	2.34	0.35
ZANZIBAR	11(1.1)	332 (1.14)	3.21	1.33	0.72	2.48	0.37
HVITTINGFOSS	10(1)	871 (2.99)	1.14	0.51	0.27	0.97	0.04
STANLEY	10(1)	220 (0.76)	4.35	2.25	1.17	4.31	0.02
AGONA	9 (0.9)	419 (1.44)	2.10	0.93	0.47	1.83	0.84
MONTEVIDEO	9 (0.9)	162 (0.56)	5.26	2.55	1.28	5.09	0.01
PARATYPHI B JAVA	9 (0.9)	346 (1.19)	2.54	1.33	0.67	2.61	0.41
DUBLIN	8 (0.8)	13 (0.04)	38.1	26.93	10.66	68.02	<0.001
READING	8 (0.8)	329 (1.13)	2.37	1.17	0.57	2.40	0.67
ANATUM	7 (0.7)	299 (1.03)	2.29	0.98	0.45	2.10	0.95
INFANTIS	6 (0.6)	248 (0.85)	2.36	1.05	0.46	2.40	0.90
MISSISSIPPI	6 (0.6)	75 (0.26)	7.41	3.21	1.36	7.53	0.01
NEWPORT	6 (0.6)	154 (0.53)	3.73	1.92	0.84	4.41	0.12
BOVISMORBIFICANS	5 (0.5)	224 (0.77)	2.18	0.95	0.39	2.34	0.91
BREDENEY	5 (0.5)	36 (0.12)	12.2	6.48	2.49	16.88	<0.001
ORANIENBURG	5 (0.5)	83 (0.28)	5.68	2.81	1.12	7.04	0.03
PANAMA	5 (0.5)	16 (0.05)	23.81	15.26	5.43	42.86	<0.001
SINGAPORE	5 (0.5)	108 (0.37)	4.42	2.11	0.85	5.25	0.11
CHOLERAESUIS	4 (0.4)	2 (0.01)	66.67	77.78	13.21	457.97	<0.001
ORIENTALIS	4 (0.4)	171 (0.59)	2.29	0.91	0.33	2.49	0.86
POTSDAM	4 (0.4)	292 (1.00)	1.35	0.56	0.21	1.52	0.25
SUBSPECIES 4	4 (0.4)	55 (0.19)	6.78	3.09	1.10	8.69	0.03
THOMPSON	4 (0.4)	154 (0.53)	2.53	1.19	0.43	3.25	0.74
WELIKADE	4 (0.4)	151 (0.52)	2.58	1.18	0.43	3.24	0.75
Other^‡^	50 (4.99)	3874 (13.30)					
**Total**^§^	**1001**	**29120**					

*Proportion of invasive isolates to the total number of isolates recovered.

^†^Odds ratio of invasiveness compared to serotype Typhimurium adjusted for gender and age groups.

^**§**^Includes 13 iNTS cases which were infected with two different serotypes, 369 non-iNTS cases infected with 2 different serotypes and 4 non-iNTS cases infected with 3 different serotypes. Seven iNTS and 2221 non-iNTS single isolates had missing information on serotype.

^**‡**^Includes 30 remaining iNTS and 125 non-iNTS serotypes

In the specimen analysis, 945 (91.7%) of 1,031 isolates came from blood with the proportions of remaining specimens depicted in S5 Table. The characteristics of iNTS cases with recurrent infection and of those infected by multiple serotypes are summarized in S6 and S7 Tables.

**Fig 2:**
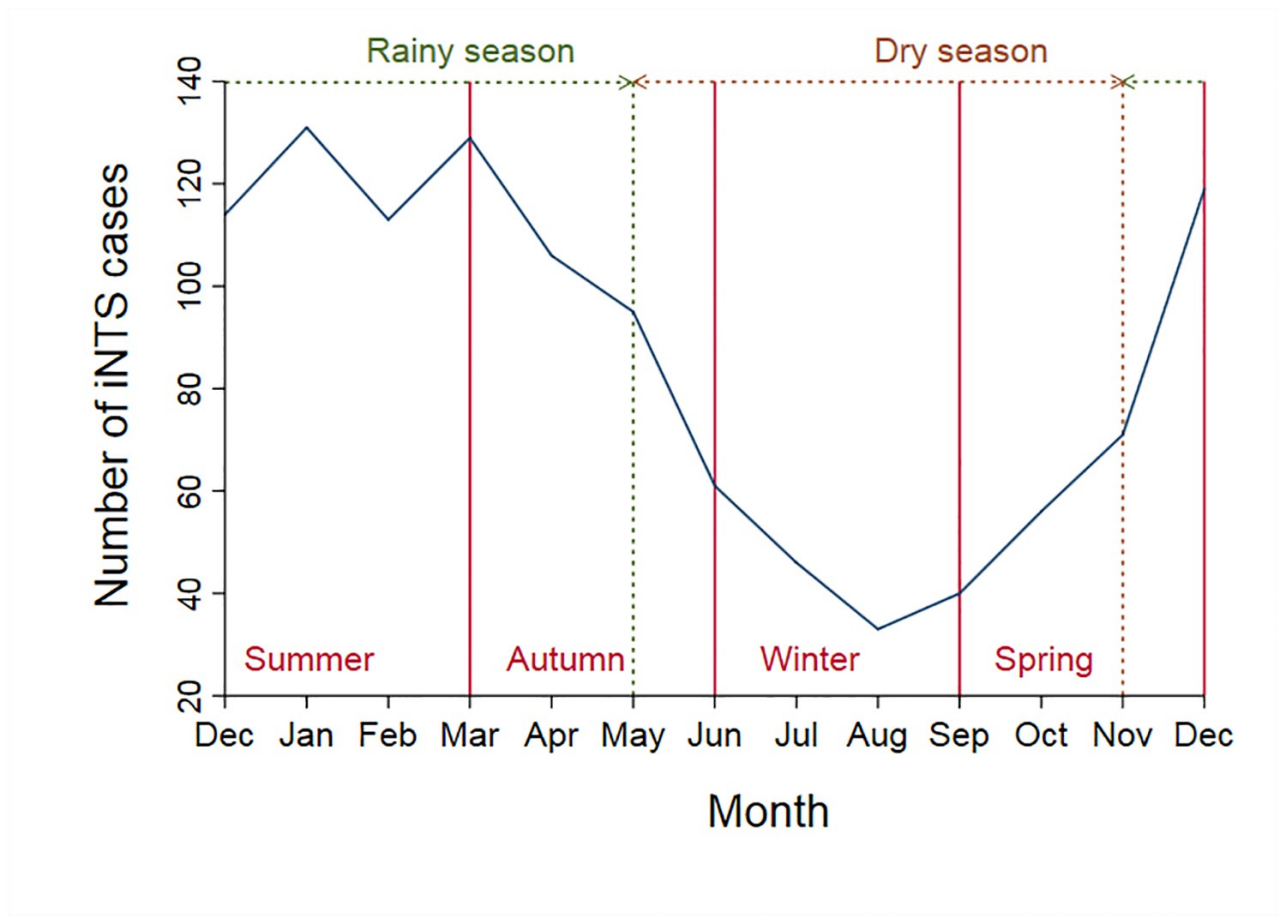
Number of iNTS cases in Queensland by month, 2007–2016.

**Fig 3:**
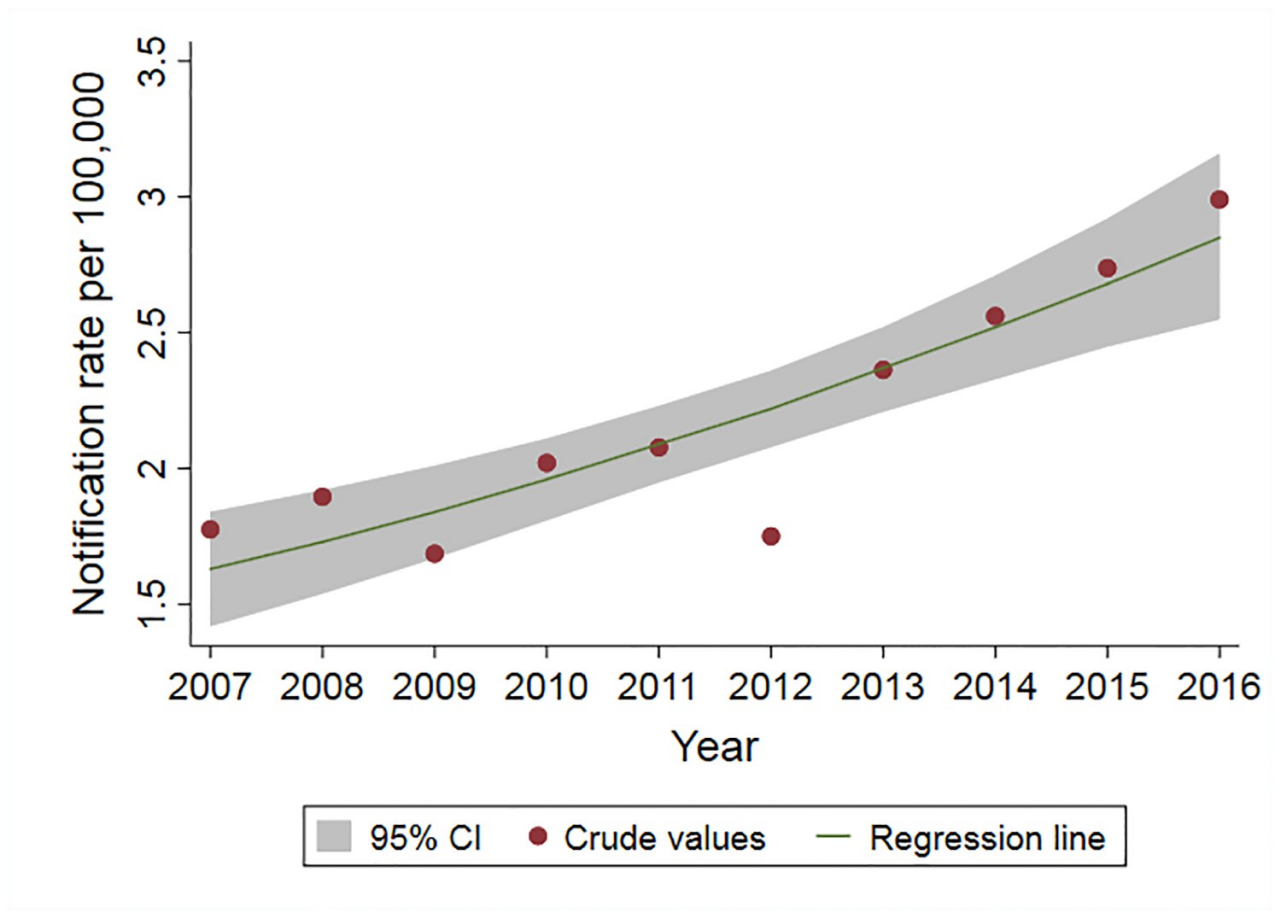
Crude and adjusted notification rates of iNTS disease in Queensland, 2007–2016.

**Fig 4:**
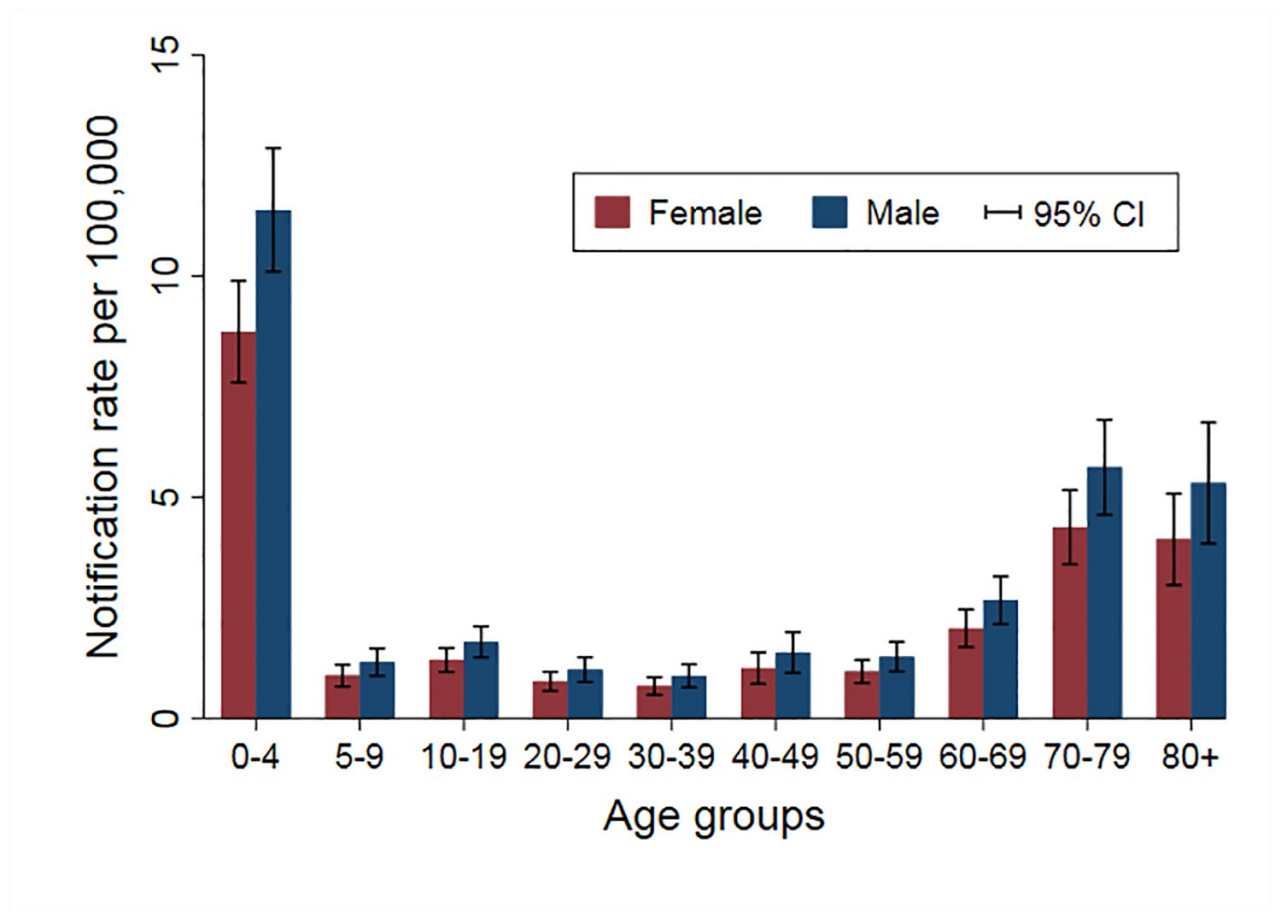
Adjusted notification rates of iNTS disease in Queensland by gender and age group, 2007–2016.

**Fig 5:**
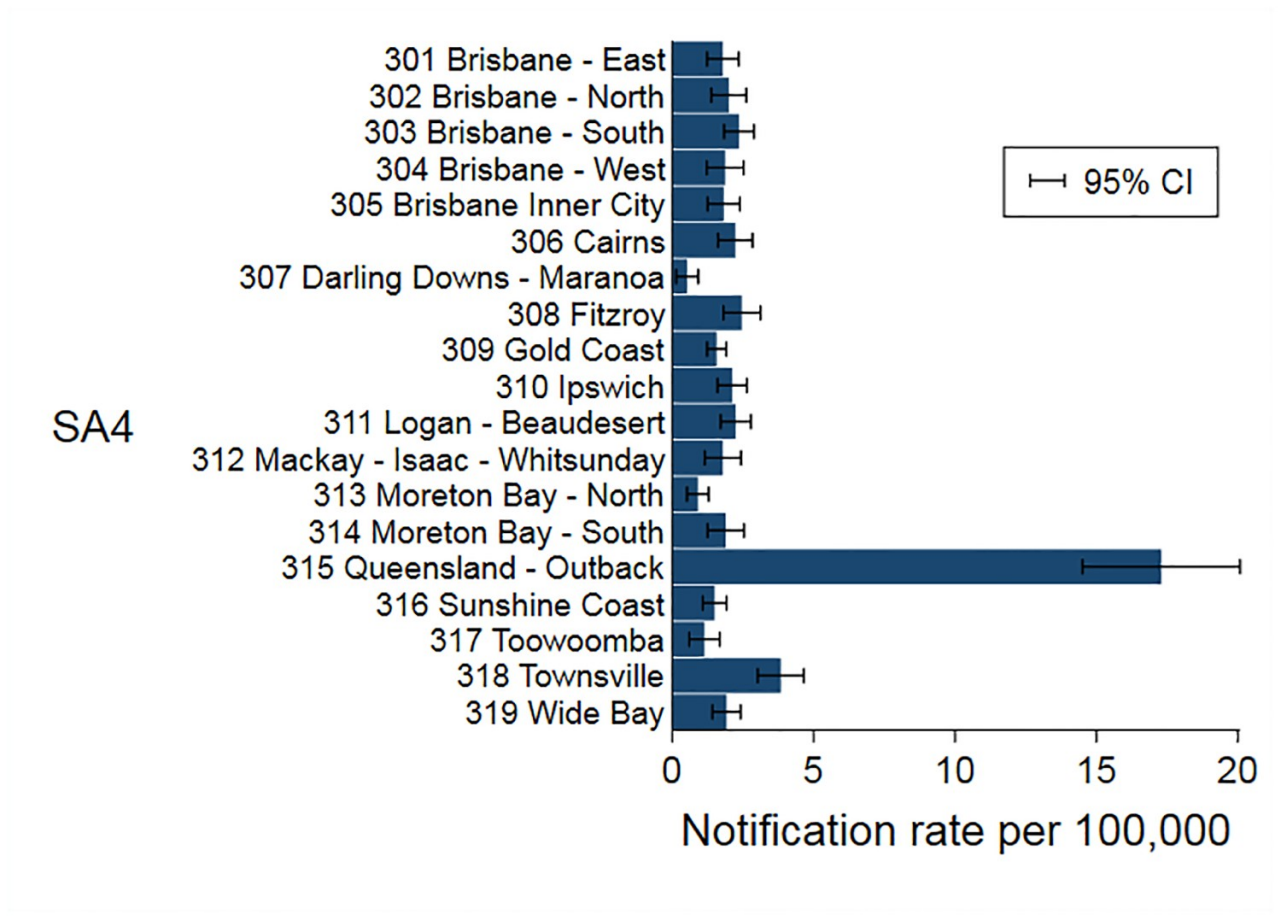
Adjusted notification rates of iNTS disease in Queensland by Statistical Area level 4, 2007–2016.

Of 995 iNTS cases included in the IRR analysis, 567 (57%) were males. The mean (range) age was 34 (0–95) years, with a significant difference in age between females and males (31. vs. 35 years, respectively; p<0.04). Of iNTS cases, 363 (36%) of 995 occurred in summer and 329 (33%) in autumn (Fig 2). The crude annual notification rate was lowest in 2012 at 1.8 per 100,000 and increased to 3.0 per 100,000 in 2016 (Fig 3). When adjusted for age group, gender, and statistical area, there was an annual 6.5% increase in the number of iNTS cases (95% CI 1.04–1.09; p<0.001) (Table 2). Crude IRR of iNTS peaked among infants (age group <1 years) for both males and females with values as high as 32 and 29 cases per 100,000 persons, respectively. The IRR was also high among young children (1–4 years old) and older adults (≥70 years old), although notification rates among older adults were much lower than in infants (Fig 4). Geographically, notifications rates ranged between 0.6 (Darling Downs—Maranoa; SA4 307) and 17.6 (Queensland—Outback; SA4 315) cases per 100,000 persons (Fig 5).

**Table 2:** Adjusted incidence rate ratios calculated using Poisson regression of iNTS disease in Queensland by sex, age, location, and year, 2007–2016.

	IRR*	95% CI^†^		P-value
**Gender (reference = female)**				
Male	1.314778	1.16	1.49	<0.001
**Age groups (reference = 30–39 years)**				
0–4	10.46591*	7.96	13.76	<0.001
5–9	1.357503	0.91	2.02	0.13
10–19	1.155499	0.82	1.63	0.41
20–29	1.579273	1.15	2.17	0.01
40–49	0.8737139	0.60	1.26	0.47
50–59	1.26938	0.90	1.79	0.18
60–69	2.433061	1.77	3.35	<0.001
70–79	5.172689	3.79	7.05	<0.001
80+	4.844231	3.40	6.90	<0.001
**SA4 (reference = 301 Brisbane—East)**				
302 Brisbane—North	1.118061	0.72	1.74	0.62
303 Brisbane South	1.316775	0.89	1.94	0.16
304 Brisbane—West	1.044321	0.65	1.67	0.86
305 Brisbane Inner City	1.017825	0.65	1.59	0.94
306 Cairns	1.24689	0.82	1.89	0.30
307 Darling Downs—Maranoa	0.2956672	0.13	0.66	<0.01
308 Fitzroy	1.375837	0.91	2.08	0.13
309 Gold Coast	0.8774511	0.60	1.28	0.50
310 Ipswich	1.185018	0.80	1.76	0.40
311 Logan—Beaudesert	1.252945	0.84	1.86	0.26
312 Mackay—Isaac—Whitsunday	1.001692	0.62	1.61	0.99
313 Moreton Bay—North	0.5057448	0.30	0.86	0.01
314 Moreton Bay—South	1.057549	0.67	1.68	0.81
315 Queensland—Outback	9.660672	6.79	13.75	<0.001
316 Sunshine Coast	0.8387001	0.55	1.28	0.41
317 Toowoomba	0.6356508	0.36	1.12	0.12
318 Townsville	2.143418*	1.47	3.13	<0.001
319 Wide Bay	1.071832	0.71	1.61	0.74
**Year (2007–2016)**	1.064551	1.04	1.09	<0.001

*Adjusted incidence rate ratio

^†^Confidence interval

Of 65 different serotypes identified in Queensland, 39 (60%) were found in outback Queensland. Among 151 isolates in outback Queensland, 33 (22.2%) were *Salmonella* serotypes Virchow, 28 (18.5%) Typhimurium, and 13 (8.6%) Aberdeen (S1 Table). Gender and age distributions of iNTS in outback Queensland were similar to other locations, with most iNTS cases being male (56%) and infants representing the most common age group (20.8%). Crude and predicted notification rates by year, gender, age group, and statistical area are presented in S8–S10 Tables.

## Discussion

We analysed 10 years of Queensland passive surveillance data to identify trends in the reported incidence and distribution of infecting *Salmonella* serotypes causing invasive disease. Males, people living in remote areas, and children had elevated incidence of iNTS disease. Higher rates of iNTS disease in remote areas were driven by an overall high number of NTS infections. However, high rates in males, infants, and elderly remained significant predictors for iNTS disease.

There are over 2,500 NTS serotypes worldwide with the great majority of infections caused by *Salmonella* Enteritidis [25]. In Australia, *Salmonella* Typhimurium is the most common serotype, as *Salmonella* Enteritidis is not endemic in egg layer flocks and is mostly acquired abroad [26]. In our study, *Salmonella* Virchow followed by *Salmonella* Typhimurium were the most common serotypes causing invasive disease in Queensland. Similarly, a study that reviewed laboratory records in a northern Queensland town over 1978–1988 also showed high proportions of *Salmonella* Virchow, accounting for 46% of all *Salmonella* bacteremias [27]. In another Australian states and territories, *Salmonella* Virchow was second to *Salmonella* Typhimurium as the most common iNTS pathogen comprising 11% and 13% of all iNTS isolates [11,12]. *Salmonella* Virchow is rare in the United States [28] but has emerged as an important pathogen in Europe [29], and Israel [30–32]. In Australia, *Salmonella* Virchow has been found in chickens, comprising 3.8% of all chicken NTS isolates; whereas bovine, porcine, and raw meat do not seem to be a common source of this serotype [33]. Surveys of vertebrates and reptiles in Queensland have found *Salmonella* Virchow in wallabies, kangaroos, and Asian house geckos [34,35].

The majority of iNTS cases occurred in summer and autumn which mostly corresponds to the wet season (November—April) in Queensland. As evident from our analyses, the seasonality of iNTS disease was driven by an overall increase in NTS cases. This is consistent with the findings of a study evaluating the effect of climate variation on *Salmonella* infection in subtropical and tropical regions in Queensland where increases in both maximum and minimum temperatures were associated with an increase in *Salmonella* infections [36].

Disease forms differ substantially by serotype, with serotypes Dublin and Choleraesuis having the highest invasiveness index [3,37,38]. This could be due to these serotypes being either more invasive or less diarrheagenic. Our results are consistent with other studies [12,37,38], although we identified very few *Salmonella* Choleraesuis and *Salmonella* Dublin infections. As previously described [2,3], infants, young children, and the elderly were most likely to develop invasive disease when compared to a referent category of 30–39 year olds. When looking at serotype distribution in specific age categories, *Salmonella* Virchow was more prevalent in infants and young children, whereas *Salmonella* Typhimurium was more common in older adults. Similar results were reported by a study from Greece that identified *Salmonella* Virchow as the most common serotype in children under one year of age [39]. As previously reported [12], being male was a significant predictor for invasive disease. Higher iNTS incidence in males was also found in another multi-national population-based cohort study that speculated that higher incidence in males might be a consequence of agricultural exposure or diet [40]. In contrast, studies investigating all NTS isolates in Australia found higher incidence in women [8].

Our study demonstrated high incidence rates of iNTS in outback Queensland. The outback is a very remote area of 1.18 million km^2^, which makes up about 68% of the land area of the state [41], and is largely (88%) agricultural [42]. Outback Queensland has a population of 79,700 with a density of 0.1 person per km^2^, the lowest of all SA4s in Queensland [43]. Our finding is in line with previous studies which identified considerably higher incidence of iNTS in rural areas when compared to urban areas [44,45]. However, when investigating the proportion of invasive and non-invasive NTS cases by statistical area, no significant difference was observed for outback Queensland (OR, 1.01; 95% CI, 0.71–1.43) suggesting that high incidence in this area is driven by an overall higher number of NTS cases.

There are several potential reasons for substantially higher rates of NTS in outback Queensland. Higher rates of NTS infection in remote areas may be linked to socio-economic status and poverty, which may mediate risk through malnutrition and comorbidities. We did not have Indigenous status in our dataset. However, outback Queensland has a higher proportion of people who identify as Aboriginal and/or Torres Strait Islander (26,560 persons) which represents 33.3% of the region’s total population [43]. Aboriginal and Torres Strait Islander people living in remote areas often live in conditions that may pose risk factors for iNTS, including poor housing conditions, food insecurity, chronic diseases, and co-infections [46–49].

While this is a large population-based study with a clearly defined denominator, there are several limitations. As we were relying only on passively collected data for surveillance, incidence of NTS infections is almost certainly underestimated, especially in remote and rural areas where access to health care is very limited [50]. As iNTS disease is more severe than non-invasive NTS and requires urgent medical attention to prevent death, selective reporting bias could be present. In addition to not having information on Aboriginal and Torres Strait Islander status, we also lacked information on comorbidities, and immunocompromising conditions that would enable us to analyze associations with high disease incidence in specific ethnic groups and statistical areas. We were not able to analyze our data by socio-economic status as Socio-Economic Indexes for Areas were unavailable at the same geographical level in which we conducted our analyses (SA4). Finally, we did not have information on travel abroad or interstate. However, a study estimating foodborne illness in Australia circa 2010 found that approximately 15% of *Salmonella* notifications were travel-associated [51].

Other investigators have used whole-genome sequencing and PCR to distinguish recrudescence from reinfection [52]. However, these methods are not included in routinely collected data in Australia. One study conducted in sub-Saharan Africa concluded that 78% of recurrences were caused by recrudescence with similar or identical *Salmonella* isolates, and reinfection accounted only for 22% of recurrences [52]. In our study, only two (0.2%) of 995 cases with iNTS disease had recurrent infection caused by the same serotype (*Salmonella* Typhimurium and *Salmonella* Chester). As we did not have molecular subtyping information on the isolates, our division was based on the time difference between two episodes of salmonellosis. As both individuals had two episodes of iNTS disease more than 30 days apart (6 months and 10 months), we considered them as reinfection and as such we counted them as separate cases of iNTS disease. However, due to such a small number of recurrent infections, this does not represent a major limitation to our study.

Our study provides an important insight into the epidemiology of iNTS disease in Australia. High rates of iNTS among males, infants, and elderly require further investigation of spatial clusters of disease, and clarification of patient and household risk factors through the conduct of case-control studies. In addition, there is a particular need to investigate and control food, animal, and environmental sources of *Salmonella* Virchow, and conduct further molecular and genomic analysis of invasive *Salmonella* Virchow isolates. Lastly, more studies are needed to update information on the global burden of iNTS disease including risk factors, predominant sources of infection, modes of transmission, and antimicrobial resistance profiles.

## Supporting information

S1 Table Proportion of serotypes by statistical areas. (DOCX)

S2 Table Distribution of *Salmonella* Virchow phage types. (DOCX)

S3 Table Distribution of *Salmonella* Typhimurium phage types. (DOCX)

S4 Table Crude and adjusted odds ratio for invasiveness of NTS. (DOCX)

S5 Table Distribution of iNTS specimens. (DOCX)

S6 Table Characteristics of individuals infected with two different iNTS serotypes. (DOCX)

S7 Table Characteristics of individuals with recurrent iNTS disease. (DOCX)

S8 Table Crude and adjusted incidence rate of iNTS disease by year. (DOCX)

S9 Table Crude and adjusted incidence rate of iNTS disease by gender and age group. (DOCX)

S10 Table Crude and adjusted incidence rate of iNTS disease by SA4. (DOCX)
