# Supplementary material for: Increasing incidence of invasive nontyphoidal Salmonella infections in Queensland, Australia, 2007-2016
Source: PLoS Negl Trop Dis. 2019 Mar 18;13(3):e0007187. doi: 10.1371/journal.pntd.0007187 (PMC6422252; doi:10.1371/journal.pntd.0007187)
Supplement: S1 Table — (DOCX) [file pntd.0007187.s001.docx]

**S1 Table.** Proportion (%) of 20 most notified iNTS serotypes of total notifications included in this study for each SA4, Queensland, 2007–2016

| **Serotype** | **Statistical Area level 4 (SA4)** | | | | | | | | | | | | | | | | | | | |
| --- | --- | --- | --- | --- | --- | --- | --- | --- | --- | --- | --- | --- | --- | --- | --- | --- | --- | --- | --- | --- |
|  | **301** | **302** | **303** | **304** | **305** | **306** | **307** | **308** | **309** | **310** | **311** | **312** | **313** | **314** | **315** | **316** | **317** | **318** | **319** |  |
| VIRCHOW | 14.6 | 25 | 17.3 | 18.2 | 34.2 | 30.2 | 0 | 49.1 | 6 | 27.7 | 28.6 | 40 | 14.3 | 21.9 | 21.9 | 28 | 12.5 | 41.7 | 24.6 |  |
| TYPHIMURIUM | 19.5 | 27.5 | 33.3 | 30.3 | 23.7 | 13.2 | 28.6 | 5.5 | 30.1 | 27.7 | 28.6 | 10 | 14.3 | 21.9 | 18.5 | 16 | 12.5 | 4.8 | 17.5 |  |
| ABERDEEN | 12.2 | 2.5 | 6.7 | 6.1 | 2.6 | 11.3 | 14.3 | 1.8 | 4.8 | 4.6 | 10 | 3.3 | 9.5 | 12.5 | 8.6 | 2 | 6.3 | 4.8 | 21.1 |  |
| ENTERITIDIS | 9.8 | 0 | 2.7 | 18.2 | 2.6 | 1.89 | 0 | 3.6 | 8.4 | 0 | 0 | 3.3 | 4.8 | 0 | 4.6 | 12 | 6.3 | 6 | 1.8 |  |
| SAINTPAUL | 9.8 | 0 | 5.3 | 3 | 0 | 1.89 | 14.3 | 3.6 | 2.4 | 0 | 0 | 10 | 0 | 3.1 | 4.6 | 6 | 0 | 7.1 | 10.5 |  |
| CHESTER | 0 | 5 | 2.7 | 6.1 | 2.6 | 1.89 | 0 | 1.8 | 1.2 | 4.6 | 4.3 | 10 | 4.8 | 3.1 | 6 | 0 | 12.5 | 4.8 | 0 |  |
| BIRKENHEAD | 4.9 | 7.5 | 6.7 | 0 | 0 | 0 | 0 | 0 | 12.1 | 6.2 | 2.9 | 0 | 4.8 | 0 | 1.3 | 6 | 0 | 1.2 | 0 |  |
| WAYCROSS | 2.4 | 0 | 0 | 0 | 5.3 | 0 | 0 | 1.8 | 14.5 | 0 | 2.9 | 13.3 | 19.1 | 0 | 2 | 0 | 6.25 | 0 | 0 |  |
| MGULANI | 0 | 0 | 0 | 0 | 0 | 3.77 | 0 | 7.3 | 0 | 0 | 0 | 0 | 4.8 | 0 | 3.3 | 0 | 0 | 7.1 | 0 |  |
| JAVIANA | 2.4 | 10 | 4 | 3 | 5.3 | 3.77 | 0 | 0 | 0 | 0 | 0 | 0 | 0 | 3.1 | 1.3 | 2 | 0 | 0 | 1.8 |  |
| CORVALLIS | 2.4 | 2.5 | 2.7 | 3 | 0 | 1.89 | 0 | 0 | 1.2 | 3.1 | 1.4 | 3.3 | 0 | 0 | 0 | 6 | 0 | 0 | 3.5 |  |
| HEIDELBERG | 0 | 2.5 | 1.3 | 0 | 2.6 | 1.89 | 0 | 5.5 | 2.4 | 0 | 0 | 0 | 0 | p | 2 | 2 | 0 | 2.4 | 0 |  |
| SUBSPECIES 1 | 4.9 | 2.5 | 1.3 | 0 | 0 | 3.77 | 14.3 | 0 | 0 | 1.5 | 2.9 | 0 | 0 | 3.1 | 1.3 | 0 | 0 | 0 | 0 |  |
| ZANZIBAR | 0 | 2.5 | 0 | 0 | 0 | 0 | 0 | 0 | 1.2 | 1.5 | 1. | 3.3 | 0 | 0 | 1.3 | 0 | 0 | 2.4 | 3.5 |  |
| HVITTINGFOSS | 0 | 0 | 0 | 0 | 0 | 3.77 | 0 | 3.6 | 0 | 0 | 0 | 0 | 0 | 3.1 | 1.3 | 2 | 0 | 0 | 3.5 |  |
| STANLEY | 0 | 0 | 0 | 0 | 2.6 | 1.89 | 0 | 0 | 3.6 | 0 | 2.9 | 0 | 0 | 3.1 | 0.7 | 0 | 0 | 1.2 | 0 |  |
| AGONA | 0 | 0 | 1.3 | 0 | 0 | 0 | 0 | 0 | 0 | 1.5 | 0 | 0 | 0 | 6.3 | 2.7 | 2 | 0 | 0 | 0 |  |
| MONTEVIDEO | 4.9 | 2.5 | 0 | 0 | 0 | 0 | 0 | 0 | 0 | 1.5 | 0 | 0 | 0 | 6.3 | 1.3 | 0 | 0 | 0 | 1.8 |  |
| PARATYPHI B JAVA | 0 | 2.5 | 1.3 | 0 | 2.6 | 0 | 0 | 1.8 | 0 | 0 | 2.9 | 0 | 4.8 | 0 | 0.7 | 0 | 6.25 | 0 | 0 |  |
| DUBLIN | 0 | 0 | 1.3 | 0 | 0 | 0 | 14.3 | 1.8 | 0 | 0 | 0 | 0 | 9.5 | 0 | 1.3 | 2 | 0 | 0 | 0 |  |

**Notes:**

SA4: 301, Brisbane – East; 302, Brisbane – North; 303, Brisbane – South; 304, Brisbane – West; 305, Brisbane Inner City; 306, Cairns; 307, Darling Downs – Maranoa; 308, Fitzroy; 309, Gold Coast; 310, Ipswich; 311, Logan - Beaudesert; 312, Mackay – Isaac – Whitsunday; 313, Moreton Bay – North; 314, Moreton Bay – South; 315, Queensland – Outback; 316, Sunshine Coast; 317, Toowoomba; 318, Townsville, 319, Wide Bay.
