# Supplementary material for: Increasing incidence of invasive nontyphoidal Salmonella infections in Queensland, Australia, 2007-2016
Source: PLoS Negl Trop Dis. 2019 Mar 18;13(3):e0007187. doi: 10.1371/journal.pntd.0007187 (PMC6422252; doi:10.1371/journal.pntd.0007187)
Supplement: S2 Table — (DOCX) [file pntd.0007187.s002.docx]

**S2 Table.** Distribution of *Salmonella* Virchow phage types causing invasive disease in Queensland, 2007-2016

| **Subtype** | **Frequency** | **%** |
| --- | --- | --- |
| PT 8 | 59 | 58.42 |
| PT 25 VAR 1 | 10 | 9.9 |
| PT 34 | 8 | 7.92 |
| PT 25 | 6 | 5.94 |
| PT 32 | 6 | 5.94 |
| PT 17 | 2 | 1.98 |
| PT 43 | 2 | 1.98 |
| PT 45 | 2 | 1.98 |
| PT 15 | 1 | 0.99 |
| PT 25A | 1 | 0.99 |
| PT 31 | 1 | 0.99 |
| PT 34 VAR 2 | 1 | 0.99 |
| PT 45B VAR 1 | 1 | 0.99 |
| PT RDNC | 1 | 0.99 |
| Total | 101 | 100 |

**Notes:**

153 cases had missing information on subtype.
