# Supplementary material for: Increasing incidence of invasive nontyphoidal Salmonella infections in Queensland, Australia, 2007-2016
Source: PLoS Negl Trop Dis. 2019 Mar 18;13(3):e0007187. doi: 10.1371/journal.pntd.0007187 (PMC6422252; doi:10.1371/journal.pntd.0007187)
Supplement: S3 Table — (DOCX) [file pntd.0007187.s003.docx]

**S3 Table.** Distribution of *Salmonella* Typhimurium phage types causing invasive disease in Queensland, 2007-2016

| **Subtype** | **Frequency** | **%** |
| --- | --- | --- |
| PT 135A | 16 | 16.33 |
| PT 170 | 11 | 11.22 |
| PT 9 | 11 | 11.22 |
| PT 197 | 9 | 9.18 |
| PT 135 | 7 | 7.14 |
| PT 44 | 6 | 6.12 |
| PT 3 | 4 | 4.08 |
| PT RDNC | 4 | 4.08 |
| PT 193 | 3 | 3.06 |
| PT U302 | 3 | 3.06 |
| PT U307 | 3 | 3.06 |
| PT 12A | 2 | 2.04 |
| PT 141 | 2 | 2.04 |
| PT 179 | 2 | 2.04 |
| PT 29 | 2 | 2.04 |
| PT 136 | 1 | 1.02 |
| PT 141 VAR 2 | 1 | 1.02 |
| PT 150 | 1 | 1.02 |
| PT 186 | 1 | 1.02 |
| PT 4 | 1 | 1.02 |
| PT 43 | 1 | 1.02 |
| PT 6 VAR 1 | 1 | 1.02 |
| PT 60 | 1 | 1.02 |
| PT 7 VAR 1 | 1 | 1.02 |
| PT RDNC A035 | 1 | 1.02 |
| PT RDNC A062 | 1 | 1.02 |
| PT RDNC A064 | 1 | 1.02 |
| PT U290 | 1 | 1.02 |
| Total | 98 | 100 |

**Notes:**

105 cases had missing information on subtype.
