# Supplementary material for: Increasing incidence of invasive nontyphoidal Salmonella infections in Queensland, Australia, 2007-2016
Source: PLoS Negl Trop Dis. 2019 Mar 18;13(3):e0007187. doi: 10.1371/journal.pntd.0007187 (PMC6422252; doi:10.1371/journal.pntd.0007187)
Supplement: S4 Table — (DOCX) [file pntd.0007187.s004.docx]

**S4 Table.** Crude and adjusted odds ratio for invasiveness of NTS in Queensland, 2007-2016

|  | **OR** | **95% CI*** | | **P-value** | **aOR**^†^ | **95% CI*** | | **P-value** |
| --- | --- | --- | --- | --- | --- | --- | --- | --- |
| **Gender (reference = female)** |  |  |  |  |  |  |  |  |
| Male | 1.35 | 1.19 | 1.53 | <0.001 | 1.32 | 1.16 | 1.50 | <0.001 |
| **Age groups (reference = 30-39)** |  |  |  |  |  |  |  |  |
| age<1 | 1.92 | 1.44 | 2.58 | <0.001 | 1.41 | 1.04 | 1.91 | 0.03 |
| 1-4 | 1.17 | 0.86 | 1.58 | 0.32 | 0.91 | 0.66 | 1.24 | 0.55 |
| 5-9 | 0.99 | 0.67 | 1.48 | 0.97 | 0.93 | 0.62 | 1.40 | 0.73 |
| 10-19 | 1.63 | 1.15 | 2.32 | 0.01 | 1.59 | 1.10 | 2.27 | 0.01 |
| 20-29 | 1.39 | 1.01 | 1.91 | 0.04 | 1.38 | 0.99 | 1.91 | 0.06 |
| 40-49 | 0.93 | 0.64 | 1.35 | 0.69 | 0.88 | 0.60 | 1.28 | 0.50 |
| 50-59 | 1.31 | 0.93 | 1.85 | 0.12 | 1.31 | 0.92 | 1.87 | 0.13 |
| 60-69 | 2.09 | 1.51 | 2.88 | <0.001 | 2.04 | 1.46 | 2.84 | <0.001 |
| 70-79 | 4.04 | 2.95 | 5.54 | <0.001 | 4.12 | 2.97 | 5.71 | <0.001 |
| 80+ | 3.39 | 2.35 | 4.88 | <0.001 | 3.69 | 2.53 | 5.39 | <0.001 |
| **Season (reference = spring)** |  |  |  |  |  |  |  |  |
| Summer | 1.19 | 0.99 | 1.43 | 0.07 |  |  |  |  |
| Autumn | 1.16 | 0.96 | 1.41 | 0.11 |  |  |  |  |
| Winter | 1.06 | 0.84 | 1.33 | 0.63 |  |  |  |  |
| **SA4 (reference = 301 Brisbane - East)** |  |  |  |  |  |  |  |  |
| 302 Brisbane - North | 1.04 | 0.67 | 1.62 | 0.85 |  |  |  |  |
| 303 Brisbane South | 1.15 | 0.78 | 1.69 | 0.48 |  |  |  |  |
| 304 Brisbane - West | 0.94 | 0.59 | 1.49 | 0.79 |  |  |  |  |
| 305 Brisbane Inner City | 0.87 | 0.56 | 1.36 | 0.54 |  |  |  |  |
| 306 Cairns | 1.32 | 0.87 | 1.99 | 0.19 |  |  |  |  |
| 307 Darling Downs - Maranoa | 0.46 | 0.21 | 1.04 | 0.06 |  |  |  |  |
| 308 Fitzroy | 1.31 | 0.87 | 1.98 | 0.20 |  |  |  |  |
| 309 Gold Coast | 1.03 | 0.70 | 1.50 | 0.89 |  |  |  |  |
| 310 Ipswich | 1.31 | 0.88 | 1.95 | 0.19 |  |  |  |  |
| 311 Logan - Beaudesert | 1.23 | 0.83 | 1.82 | 0.30 |  |  |  |  |
| 312 Mackay - Isaac - Whitsunday | 0.66 | 0.41 | 1.06 | 0.08 |  |  |  |  |
| 313 Moreton bay - North | 1.96 | 1.15 | 3.37 | 0.01 |  |  |  |  |
| 314 Moreton Bay - South | 0.96 | 0.61 | 1.53 | 0.88 |  |  |  |  |
| 315 Queensland - Outback | 1.01 | 0.71 | 1.43 | 0.97 |  |  |  |  |
| 316 Sunshine Coast | 1.04 | 0.69 | 1.58 | 0.85 |  |  |  |  |
| 317 Toowoomba | 1.05 | 0.59 | 1.86 | 0.88 |  |  |  |  |
| 318 Townsville | 1.04 | 0.71 | 1.51 | 0.85 |  |  |  |  |
| 319 Wide Bay | 1.19 | 0.79 | 1.79 | 0.41 |  |  |  |  |
| **Year (reference=2007)** |  |  |  |  |  |  |  |  |
| 2008 | 1.30 | 0.94 | 1.79 | 0.11 |  |  |  |  |
| 2009 | 0.98 | 0.70 | 1.36 | 0.89 |  |  |  |  |
| 2010 | 1.03 | 0.75 | 1.41 | 0.84 |  |  |  |  |
| 2011 | 1.05 | 0.77 | 1.43 | 0.78 |  |  |  |  |
| 2012 | 0.91 | 0.66 | 1.26 | 0.59 |  |  |  |  |
| 2013 | 1.17 | 0.86 | 1.57 | 0.31 |  |  |  |  |
| 2014 | 0.81 | 0.60 | 1.08 | 0.15 |  |  |  |  |
| 2015 | 0.78 | 0.59 | 1.05 | 0.10 |  |  |  |  |
| 2016 | 0.97 | 0.73 | 1.29 | 0.84 |  |  |  |  |

**Notes:**

* Confidence interval

^†^ Adjusted for gender, age group, and serotype.
