# Supplementary material for: Increasing incidence of invasive nontyphoidal Salmonella infections in Queensland, Australia, 2007-2016
Source: PLoS Negl Trop Dis. 2019 Mar 18;13(3):e0007187. doi: 10.1371/journal.pntd.0007187 (PMC6422252; doi:10.1371/journal.pntd.0007187)
Supplement: S5 Table — (DOCX) [file pntd.0007187.s005.docx]

**S5 Table.** Distribution of iNTS specimens in Queensland, 2007-2016

| **Specimen** | **Frequency** | **%** |
| --- | --- | --- |
| Blood | 945 | 91.66 |
| Cerebrospinal fluid | 10 | 0.97 |
| Peritoneal fluid | 16 | 1.55 |
| Pleural Fluid | 5 | 0.48 |
| Other fluid/tissue | 55 | 5.33 |
| **Total** | **1,031** | **100** |

**Notes:**

Table includes 34 cases which were culture positive for two different specimens and one case which had positive three different specimens. Two cases had recurrent infection.
