# Supplementary material for: Increasing incidence of invasive nontyphoidal Salmonella infections in Queensland, Australia, 2007-2016
Source: PLoS Negl Trop Dis. 2019 Mar 18;13(3):e0007187. doi: 10.1371/journal.pntd.0007187 (PMC6422252; doi:10.1371/journal.pntd.0007187)
Supplement: S6 Table — (DOCX) [file pntd.0007187.s006.docx]

**S6 Table.** Characteristics of individuals infected with two different iNTS serotypes in Queensland, 2007-2016

| **Person** | **Year** | **Age group (years)** | **Gender** | **Serotypes** |
| --- | --- | --- | --- | --- |
| 1 | 2008 | 1-4 | Female | READING, TYPHIMURIUM |
| 2 | 2010 | 1-4 | Male | VIRCHOW, ABERDEEN |
| 3 | 2014 | 1-4 | Female | CHESTER, TENNESSEE |
| 4 | 2013 | <1 | Female | BREDENEY, DUBLIN |
| 5 | 2013 | 51 | Female | VIRCHOW, INFANTIS |
| 6 | 2013 | 54 | Male | CORVALLIS, CHAILEY |
| 7 | 2014 | <1 | Female | VIRCHOW, INFANTIS |
| 8 | 2014 | 70-79 | Female | SUBSPECIES 1, TYPHIMURIUM |
| 9 | 2015 | <1 | Female | RUBISLAW, POTSDAM |
| 10 | 2015 | 20-29 | Male | CHESTER, ENTERICA |
| 11 | 2015 | 20-29 | Male | TYPHIMURIUM, ANATUM |
| 12 | 2015 | 70-79 | Female | SUBSPECIES 1, ABERDEEN |
| 13 | 2016 | 50-59 | Male | KENTUCKY, JAVIANA |

g
