# Supplementary material for: Increasing incidence of invasive nontyphoidal Salmonella infections in Queensland, Australia, 2007-2016
Source: PLoS Negl Trop Dis. 2019 Mar 18;13(3):e0007187. doi: 10.1371/journal.pntd.0007187 (PMC6422252; doi:10.1371/journal.pntd.0007187)
Supplement: S7 Table — (DOCX) [file pntd.0007187.s007.docx]

**S7 Table.** Characteristics of individuals with recurrent iNTS disease in Queensland, 2007-2016

| \| **Person** \| **Year** \| **Age group (years)** \| **Gender** \| **Serotype** \| \| --- \| --- \| --- \| --- \| --- \| \| 1 \| 2013 \| 70-79 \| Male \| CHESTER \| \|  \| 2013 \| 70-79 \| Male \| CHESTER \| \| 2 \| 2015 \| 70-79 \| Male \| TYPHIMURIUM \| \|  \| 2016 \| 70-79 \| Male \| TYPHIMURIUM \| |  |
| --- | --- | --- | --- | --- | --- | --- | --- | --- | --- | --- | --- | --- | --- | --- | --- | --- | --- | --- | --- | --- | --- | --- | --- | --- | --- | --- |
