# Supplementary material for: Increasing incidence of invasive nontyphoidal Salmonella infections in Queensland, Australia, 2007-2016
Source: PLoS Negl Trop Dis. 2019 Mar 18;13(3):e0007187. doi: 10.1371/journal.pntd.0007187 (PMC6422252; doi:10.1371/journal.pntd.0007187)
Supplement: S8 Table — (DOCX) [file pntd.0007187.s008.docx]

**S8 Table.** Crude and adjusted notification rate of iNTS disease in Queensland by year, 2007-2016

| **Year** | **iNTS cases** | **Population** | **Crude notification rate per 100,000** | **Adjusted notification rate per 100,000** | **95 % CI** |
| --- | --- | --- | --- | --- | --- |
| 2007 | 73 | 4111018 | 1.78 | 1.63 | 1.42-1.84 |
| 2008 | 80 | 4219505 | 1.90 | 1.73 | 1.54-1.92 |
| 2009 | 73 | 4328771 | 1.69 | 1.84 | 1.67-2.01 |
| 2010 | 89 | 4404744 | 2.02 | 1.96 | 1.81-2.11 |
| 2011 | 93 | 4476778 | 2.08 | 2.09 | 1.95-2.23 |
| 2012 | 80 | 4569863 | 1.75 | 2.22 | 2.08-2.36 |
| 2013 | 110 | 4654521 | 2.36 | 2.37 | 2.21-2.52 |
| 2014 | 121 | 4724417 | 2.56 | 2.52 | 2.33-2.71 |
| 2015 | 131 | 4784367 | 2.74 | 2.68 | 2.45-2.92 |
| 2016 | 145 | 4848877 | 2.99 | 2.85 | 2.55-3.16 |
