# Supplementary material for: Increasing incidence of invasive nontyphoidal Salmonella infections in Queensland, Australia, 2007-2016
Source: PLoS Negl Trop Dis. 2019 Mar 18;13(3):e0007187. doi: 10.1371/journal.pntd.0007187 (PMC6422252; doi:10.1371/journal.pntd.0007187)
Supplement: S9 Table — (DOCX) [file pntd.0007187.s009.docx]

**S9 Table.** Crude and adjusted incidence rate of iNTS disease in Queensland by gender and age group, 2007-2016

| **Gender** | **Age groups** | **iNTS cases** | **Population** | **Crude notification rate per 100,000** | **Adjusted notification rate per 100,000** | **95% CI** |
| --- | --- | --- | --- | --- | --- | --- |
| Female | <1 | 86 | 300196 | 28.65 | 8.74* | 7.59-9.89 |
|  | 1-4 | 67 | 1185094 | 5.65 |  |  |
|  | 5-9 | 22 | 1459116 | 1.51 | 1.13 | 0.78-1.49 |
|  | 10-19 | 27 | 2915163 | 0.93 | 0.97 | 0.72-1.21 |
|  | 20-29 | 35 | 3207935 | 1.09 | 1.32 | 1.05-1.59 |
|  | 30-39 | 30 | 3144829 | 0.95 | 0.84 | 0.62-1.05 |
|  | 40-49 | 25 | 3197201 | 0.78 | 0.73 | 0.53-0.93 |
|  | 50-59 | 27 | 2855665 | 0.95 | 1.06 | 0.8-1.32 |
|  | 60-69 | 34 | 2193585 | 1.55 | 2.03 | 1.61-2.46 |
|  | 70-79 | 44 | 1287297 | 3.42 | 4.32 | 3.48-5.15 |
|  | 80+ | 31 | 897566 | 3.45 | 4.05 | 3.01-5.08 |
| Male | <1 | 101 | 317070 | 31.85 | 11.49* | 10.1-12.89 |
|  | 1-4 | 70 | 1250408 | 5.60 |  |  |
|  | 5-9 | 19 | 1540996 | 1.23 | 1.49 | 1.03-1.95 |
|  | 10-19 | 40 | 3053897 | 1.31 | 1.27 | 0.96-1.58 |
|  | 20-29 | 67 | 3272908 | 2.05 | 1.73 | 1.38-2.08 |
|  | 30-39 | 31 | 3100641 | 1.00 | 1.10 | 0.82-1.38 |
|  | 40-49 | 28 | 3112965 | 0.90 | 0.96 | 0.7-1.22 |
|  | 50-59 | 42 | 2801610 | 1.50 | 1.39 | 1.06-1.73 |
|  | 60-69 | 66 | 2207182 | 3.00 | 2.67 | 2.13-3.21 |
|  | 70-79 | 74 | 1227523 | 6.03 | 5.68 | 4.6-6.75 |
|  | 80+ | 29 | 594014 | 4.88 | 5.32 | 3.95-6.69 |

**Notes:**

*Adjusted notification rate in 0-4 age group.
