# Supplementary material for: Increasing incidence of invasive nontyphoidal Salmonella infections in Queensland, Australia, 2007-2016
Source: PLoS Negl Trop Dis. 2019 Mar 18;13(3):e0007187. doi: 10.1371/journal.pntd.0007187 (PMC6422252; doi:10.1371/journal.pntd.0007187)
Supplement: S10 Table — (DOCX) [file pntd.0007187.s010.docx]

**S10 Table.** Crude and adjusted incidence rate of iNTS disease in Queensland by Statistical Area level 4 (SA4), 2007-2016

| **SA4** | **iNTS cases** | **Population** | **Crude notification rate per 100,000** | **Adjusted notification rate per 100,000** | **95% CI** |
| --- | --- | --- | --- | --- | --- |
| 301 | 39 | 2188469 | 1.78 | 1.79 | 1.23-2.35 |
| 302 | 40 | 1992243 | 2.01 | 2 | 1.38-2.62 |
| 303 | 74 | 3321875 | 2.23 | 2.36 | 1.83-2.89 |
| 304 | 32 | 1792530 | 1.79 | 1.87 | 1.22-2.52 |
| 305 | 39 | 2411571 | 1.62 | 1.82 | 1.25-2.39 |
| 306 | 52 | 2341109 | 2.22 | 2.23 | 1.62-2.84 |
| 307 | 7 | 1250517 | 0.56 | 0.53 | 0.14-0.92 |
| 308 | 54 | 2180412 | 2.48 | 2.46 | 1.81-3.12 |
| 309 | 84 | 5365221 | 1.57 | 1.57 | 1.23-1.91 |
| 310 | 64 | 2959254 | 2.16 | 2.12 | 1.60-2.64 |
| 311 | 68 | 3032946 | 2.24 | 2.24 | 1.71-2.78 |
| 312 | 30 | 1701858 | 1.76 | 1.79 | 1.15-2.43 |
| 313 | 21 | 2233297 | 0.94 | 0.91 | 0.52-1.29 |
| 314 | 33 | 1760522 | 1.87 | 1.89 | 1.25-2.54 |
| 315 | 149 | 847057 | 17.59 | 17.29 | 14.5-20.07 |
| 316 | 50 | 3243299 | 1.54 | 1.5 | 1.08-1.92 |
| 317 | 17 | 1453370 | 1.175 | 1.14 | 0.6-1.68 |
| 318 | 85 | 2244238 | 3.79 | 3.84 | 3.02-4.65 |
| 319 | 57 | 2803073 | 2.03 | 1.92 | 1.42-2.42 |

**Notes:**

SA4: 301, Brisbane – East; 302, Brisbane – North; 303, Brisbane – South; 304, Brisbane – West; 305, Brisbane Inner City; 306, Cairns; 307, Darling Downs – Maranoa; 308, Fitzroy; 309, Gold Coast; 310, Ipswich; 311, Logan - Beaudesert; 312, Mackay – Isaac – Whitsunday; 313, Moreton Bay – North; 314, Moreton Bay – South; 315, Queensland – Outback; 316, Sunshine Coast; 317, Toowoomba; 318, Townsville, 319, Wide Bay.
